# Supplementary material for: Mechanisms governing the pioneering and redistribution capabilities of the non-classical pioneer PU.1
Source: Nat Commun. 2020 Jan 21;11:402. doi: 10.1038/s41467-019-13960-2 (PMC6972792; doi:10.1038/s41467-019-13960-2)
Supplement: Supplementary file 7 — Source data [file 41467_2019_13960_MOESM7_ESM.zip › Source_Data/Figure6/Figure6B_MotifScanOutput/ETS1_mutPU.1/homerResults/motif14.similar.html]

motif14

## Information for motif14

A
C
T
G
A
C
T
G
A
T
C
G
T
A
C
G
T
C
A
G
C
T
A
G
A
C
T
G
  
Reverse Opposite:  

A
G
T
C
A
G
T
C
A
G
T
C
A
T
G
C
A
T
G
C
A
G
T
C
A
G
T
C
  

|  |  |
| --- | --- |
| p-value: | 1e-133 |
| log p-value: | -3.078e+02 |
| Information Content per bp: | 1.808 |
| Number of Target Sequences with motif | 5238.0 |
| Percentage of Target Sequences with motif | 38.84% |
| Number of Background Sequences with motif | 9544.5 |
| Percentage of Background Sequences with motif | 27.20% |
| Average Position of motif in Targets | 99.9 +/- 54.6bp |
| Average Position of motif in Background | 98.6 +/- 62.1bp |
| Strand Bias (log2 ratio + to - strand density) | -0.0 |
| Multiplicity (# of sites on avg that occur together) | 1.54 |
| Motif File: | file (matrix) reverse opposite |

### Similar de novo motifs found

|  |  |  |  |  |  |  |  |
| --- | --- | --- | --- | --- | --- | --- | --- |
| Rank | Match Score | Redundant Motif | P-value | log P-value | % of Targets | % of Background | Motif file |
| 1 | 0.756 | G A C T T A C G C A T G A T G C G A T C T G A C A G T C C A T G A G T C A G T C A G T C | 1e-116 | -267.921134 | 23.67% | 14.67% | motif file (matrix) |
| 2 | 0.857 | T A G C T A G C A G T C A T G C A T G C A T G C G A T C G A T C A G T C | 1e-101 | -234.330783 | 33.35% | 23.63% | motif file (matrix) |
| 3 | 0.822 | A G T C G A T C G A T C A G T C A T G C A G T C G A T C G A T C G T A C G A T C | 1e-87 | -202.192711 | 28.45% | 19.91% | motif file (matrix) |
